# Supramolecular nanomedicine in the intelligent cancer therapy: recent advances and future

**DOI:** 10.3389/fphar.2024.1490139

**Published:** 2024-10-11

**Authors:** Shuo Li, Yujiao Wang, Chao Li, Binghao Zhou, Xiaoxi Zeng, Hong Zhu

**Affiliations:** ^1^ Department of Thoracic Surgery, West China Hospital, Sichuan University, Chengdu, Sichuan, China; ^2^ Lung Cancer Center, West China Hospital, Sichuan University, Chengdu, Sichuan, China; ^3^ Division of Nephrology, West China Hospital, Sichuan University, Chengdu, Sichuan, China; ^4^ Second Clinical Medical College, Lanzhou University, Lanzhou, Gansu, China; ^5^ Cancer Center, West China Hospital, Sichuan University, Chengdu, Sichuan, China

**Keywords:** supramolecular nanomedicine, cancer, chemotherapy, immune therapy, photodynamic therapy, photothemal therapy, targeted therapy

## Abstract

In recent years, the incidence of cancer has been increasing year by year, and the burden of the disease and the economic burden caused by it has been worsening. Although chemotherapy, immunotherapy, targeted therapy and other therapeutic means continue to progress, they still inevitably have problems such as high toxicity and side effects, susceptibility to drug resistance, and high price. Photothermal therapy and photodynamic therapy have demonstrated considerable advantages in cancer imaging and treatment due to their minimally invasive and selective nature. However, their development has been constrained by challenges related to drug delivery. In recent times, drug delivery systems constructed based on supramolecular chemistry have been the subject of considerable interest, particularly in view of their compatibility with the high permeability and long retention effect of tumors. Furthermore, the advantage of dissociating the active ingredient under pH, light and other stimuli makes them unique in cancer therapy. This paper reviews the current status of supramolecular nanomedicines in cancer therapy, elucidating the challenges faced and providing a theoretical basis for the efficient and precise treatment of malignant tumors.

## 1 Introduction

The latest global cancer statistics report shows that there will be nearly 20 million new cancer cases globally in 2022, while cancer accounts for about guide nearly 1/6 (16.8%) of the global causes of death, and at the same time, it is responsible for a quarter (22.8%) of the deaths due to non-communicable diseases (Bray et al., 2024). Cancer is a major social, public health and economic problem of the 21st century. Despite advances in medicine and technology, which have expanded and enhanced cancer treatment modalities, current approaches such as chemotherapy, immunotherapy, and targeted therapy often lead to various toxic side effects and the development of tumor multidrug resistance over time. These years, photothermal therapy (PTT) and photodynamic therapy (PDT) based on the combination of light and light-absorbing nanomedicines is an emerging and promising cancer treatment modality. These methods offer high spatiotemporal selectivity, reduced invasiveness, and fewer side effects, and are gaining prominence in cancer therapy. However, problems like photothermal conversion efficiency and drug delivery also appear. In addition, the efficacy of some anticancer drugs is compromised by drug properties like poor solubility, stability, and targeting. Therefore, a new drug delivery and synthesis method is urgently needed to achieve efficient and precise treatment of malignant tumors.

Supramolecular nanomedicines integrate the principles of nanotechnology and supramolecular chemistry to construct complex molecular assemblies through noncovalent interactions, like hydrogen bonding, van der Waals forces, metal coordination, electrostatic interactions, and host-guest complexation (Wang et al., 2018), they show preparative feasibility, biodegradability, and stimuli-responsiveness. Enhanced permeability and retention (EPR) effect is one of the mechanisms of nanomedicine targeting, which is caused by abnormal tumor blood vessels with large endothelial gaps and incomplete structure, and by obstruction of lymphatic fluid reflux for the lack of lymphatic vessels in tumor (Nguyen et al., 2023). Therefore, SNPs can accurately target cancer cells without easily damaging normal cells, showing blood circulation stability and tumor cell targeting. Moreover, some nanomedicines are inactive in a physiological environment in the form of prodrugs and can be activated under external (ultraviolet, near-infrared radiation (NIR), etc.) or internal (pH, reactive oxygen species (ROS), enzymes, etc.) stimuli after tumor cell internalization to kill the tumor.

In this paper, we reviewed the supramolecular nanomedicines administrated in cancer therapy, to clarify the current situation and challenges, and to provide the theoretical basis for the efficient and precise treatment of malignant tumors.

## 2 Supramolecular nanomedicines in chemotherapy

Chemotherapeutic drugs are one of the key treatments in the fight against cancer, but serious toxic side effects and related drug resistance are almost unavoidable during drug administration (Agnello et al., 2019). some small-molecule drugs and nanomedicines have been developed to overcome these problems by improving their selectivity and targeted delivery (Robey et al., 2018; Wang C. et al., 2020). Nanomedicines with combinatorial drug therapy features have been widely used as an effective approach to overcome drug resistance through synergistic therapy. These nanomedicines can simultaneously deliver multiple drugs with different/identical therapeutic targets and improve efficacy while reducing side effects (Ren et al., 2019).

### 2.1 Platinum-based chemotherapy

Platinum (Pt) drugs are potent cytotoxins that induce the formation of Pt-DNA adducts through the covalent binding of Pt atoms to two neighboring purine bases in the same DNA strand, thereby blocking transcription and leading to apoptosis (Johnstone et al., 2016). About half of all cancer patients are being treated with platinum-based drugs (Yu et al., 2015). However, free platinum drugs often cause serious side effects due to interactions with plasma proteins and normal cells (Carr et al., 2018), while they are inhibited by both intrinsic and acquired resistance in cancer (Martin et al., 2008), for which researchers have worked on the development of various Pt-encapsulated supramolecular nano systems.

K. X. Liu et al. reported a simple and robust strategy for the preparation of homogeneous polymeric Pt nanocapsules from linear dendrimers by first synthesizing a polyethylene glycol (PEG)-dendrimer poly (lysine) block copolymer (PEG-G4, fourth generation) by amidating dendrimer poly (lysine) with a specific anhydride and then complexing it with cisplatin (CDDP) complexation to form PEG-G4/amide-Pt nanocapsules, which had better stability in blood due to thermodynamically stable complexation and PEGylated surfaces. When the nanocapsules reached the acidic tumor microenvironment (TME) and intracellular compartments, the amide bond tended to break and Pt release. The results showed that PEG-G4/MSA Pt nanocapsules exhibited potent antitumor activity with reduced side effects in a high-fidelity patient-derived tumor xenograft (PDX) hepatocellular carcinoma (HCC) model compared to small molecule Pt compounds (Liu K. et al., 2021). X. D. He et al. developed a new nanoscale supramolecular self-assembled Pt-TCPP-BA with Pt nanoparticles (NPs) with trans-Pt (II), tetra (4-carboxyphenyl) porphyrin (TCPP) and benzoic acid (BA) to counteract platinum-based drug resistance. It has a hydrogen peroxide-responsive dissociation behavior and can produce biologically active trans-Pt (II) and TCPP-Pt. In addition to cytotoxic effects (inter-strand crosslinks) of trans-Pt (II), TCPP-Pt can interact with DNA through groove binding, further activate the p53-dependent and cysteine 3-mediated cell apoptosis, p21-induced cell cycle arrest, and γ-H2A.X-featured DNA damage, showing high cytotoxicity in cisplatin-resistant A549 DDP cells (He et al., 2024).

In colorectal cancer (CRC), *fusobacterium nucleatum* (Fn) is considered strongly related to post-chemotherapy recurrence and chemoresistance. X. J. Yan et al. investigated novel supramolecular up-conversion NPs (SUNPs) to overcome Fn-induced chemoresistance. Lauric acid (LA), a naturally occurring fatty acid with selective antimicrobial effects against Fn, was covalently coupled with poly (amidoamine) (PAMAM) (Zhao et al., 2022), negative oxaliplatin pro-drug (OXA–COOH) electrostatically assembled with positive PAMAM (Schueffl et al., 2021), increasing drug-carrying capacity to 30.8% (Li et al., 2023; Xin et al., 2023). In addition, the *in vivo* biocompatibility of SUNPs was significantly enhanced by encapsulating PEG shells on the surface of SUNPs through host-guest supramolecular interactions to protect the positive charge of PAMAM and off-target release of OXA-COOH. SUNPs decomposed in response to azoreductase in the acidic CRC TME under 980 nm excitation, resulting in on-demand drug release at the tumor site. *In vitro* and *in vivo* studies have shown that SUNPs are biocompatible and can significantly overcome Fn-induced chemoresistance and inhibit CRC cell proliferation (Yan et al., 2024).

### 2.2 Anthracycline-based chemotherapy

Macrocyclic hosts including crown ethers, cyclodextrins, calixarene calix, pillararenes, and cucurbit [n]urils (n = 5–8) (CB [n]s) typically have hydrophobic cavities that can be used to embed guests. CB [n]s is widely used in the drug delivery systems for diseases because of excellent affinity and biocompatibility from the synergistic effect of hydrophobic interactions and ion-dipole interactions. M. T. Chen et al. developed a CB [7] based recognition motif with doxorubicin (DOX) as a guest. In the aqueous solution, CB [7]- DOX self-assembled into spherical NPs with a diameter of about 100 nm, improving the stability and solubility of DOX. CB [7]- DOX could be effectively internalized and entered into the nucleus of the tumor cells, inducing apoptosis in U87 cells and showing anticancer effect on gliomas (Chen et al., 2022).

CB [8] has a large cavity size allowing the formation of 1:1:1 ternary host-guest complexes, which makes it different from other macrocyclic hosts. H. Wu et al. used methyl viologen (MV)-modified poly (ε-caprolactone) (PCL-MV), 6-methoxy-2-naphthol-conjugated methoxy-PEG (Nap-PEG) and CB [8] as building blocks to prepare supramolecular deblocking copolymers, which self-assembled into SNPs in aqueous solution with the ability to encapsulate the hydrophobic anticancer drug DOX, thus providing supramolecular nanomedicines (SNM@DOX), which remain intact in physiological environments thanks to strong binding affinity and play 5a key role in preventing premature drug release plays a key role in preventing the premature release of the drug, with greatly increased circulation time and tumor accumulation compared to the free drug, Adriamycin hydrochloride, mainly attributed to the EPR effect (Wu et al., 2021).

In addition to cucurbiturils, the pillararenes possess well host-guest properties for their unique structure and are widely used in the preparation of amphiphilic polymers. Ding et al. developed a pH/ROS dual-sensitive pillararenes-based supramolecular polypeptide (SPP) [5] (SPP-DOX/Ce6) with long circulation retention and preferential tumor site accumulation. Specifically, pillar [5]arene-modified polypeptide (P5-PLL-DMA) by coupling 2,3-dimethylmaleic anhydride (DMA) to poly (l-lysine) (PLL), and polypeptide prodrug (P-PLL-DOX) including a ROS-cleavable thioketal (TK) linker between DOX and PLL. Subsequently, the precursor SPP-DOX was constructed from P5-PLL-DMA and P-PLL-DOX by subject-guest recognition, and then Ce6 was encapsulated to obtain the supramolecular polypeptide prodrug SPP-DOX/Ce6. At physiological pH 7.4, the negatively charged SPP-DOX/Ce6 could avoid nonspecific protein adsorption and premature drug release, enabling SPP-DOX/Ce6 higher stability and longer blood circulation. When reaching acidic TME (PH 6.5) through the EPR effect, the DMA group rapidly hydrolyzed with positive charge reversal, which significantly enhanced cell internalization and intracellular drug accumulation. Under 660 nm light irradiation, the generated ROS effectively cleaved the TK linker and released the activated DOX, resulting in an efficient antitumor effect (Ding et al., 2022).

Notably, NPs have stimuli-responsive capabilities. Non-covalent bonds are sensitive to specific TME, so that can drug accumulate and be released on demand. X. Zhang et al. designed and synthesized PCL-MV and Nap-PEG, forming amphiphile SNPs through ternary host-guest complexation between MV, Nap, and CB. Prostate-specific membrane antigen (PSMA)-617, which contains naphthalene moiety, can be inserted into the CB [8] along with MV, enabling NP the targeting ability to cancer which overexpress PSMA. Based on the molecular recognition mechanism above, the researchers prepared DOX-loaded SNPs (SNPs@DOX) and PSMA-targeted SNPs@DOX (P-SNPs@DOX), both of which could be internalized by both 22RV1 and PC3 cells, and P-SNPs@DOX shows higher uptake rate with higher cytotoxicity due to its targeting properties (Zhang X. et al., 2022). β-Cyclodextrin (β-CD) is another core-shell tecto dendrimer (CSTD) for cancer nanomedicine applications. J. L. Gong et al. prepared CSTD with β-CD modified Generation five poly (amidoamine) (G5 PAMAM) as the core and G3 PAMAM coupled with Ad and DOTA as the shell. Then CSTD was covalently modified with 1,3-propanesultone (1,3-PS) and chelated with Gadolinium (Gd) (iii) to form PCSTD-Gd, which in turn encapsulated DOX within it. With the assistance of ultrasound-targeted microbubble destruction (UTMD), the multifunctional PCSTD-Gd/DOX/miR 21i peptide was delivered into the tumor to enhance tumor penetration and enrichment for MR imaging and TNBC combination therapy (Gong J. et al., 2023).

PEG is a widely used modification agent, but the fact that the immune system develops adverse reactions caused by the presence of anti-PEG antibodies associated with the repeated use of PEG-based drugs and preparations should not be ignored. Other water-soluble polymers including poly (N-(2-hydroxypropyl) methacrylamide (PHPMA) have not observed the phenomenon (Kierstead et al., 2015). J. C. Albuquerque reported pH-responsive polymeric vesicles loaded with DOX using PHPMA_35_-b-poly [2-(diisopropylamino) ethyl methacrylate]_75_(PHPMA_35_-b-PDPA_75_) as a building block. It can maintain colloidal stability while loading 10% w/w DOX, and due to the pH-switchable property of PDPA (pKa∼6.8), DOX loading may be released abruptly mainly in the TME, improving therapeutic efficacy and inhibiting growth in the EL4 lymphoma tumor model as compared to free DOX administration (Albuquerque et al., 2021).

**FIGURE 1:**
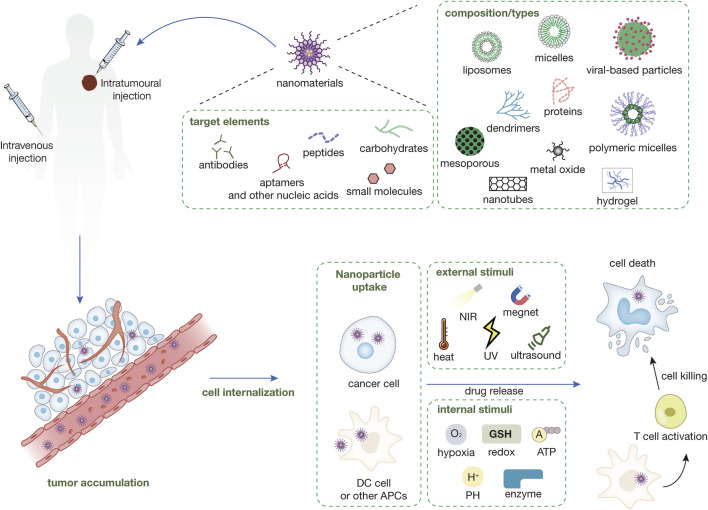
Types of nanomaterials currently in use and their surface-targeted modifications and mechanisms of nanomedicines. Nanomedicines are injected into the body either intravenously or intratumorally. Nanomedicines injected intravenously into the body need to be targeted to the tumor site through a series of mechanisms like EPR, while intratumorally injected nanomedicines can directly interact with the tumor immune microenvironment. The nanomedicine in the TME can be directly targeted into the cancer cells and release the drug and play a tumor-killing role under a variety of external or endogenous stimuli. Some nanomedicines can enter immune cells (e.g., DC cells) within the TME to enhance antigen presentation and induce immune killing of tumors.

Although self-assembled NPs have emerged as a promising way for cancer therapy, the tissue penetration of NPs is still limited due to the presence of complex biological barriers in the TME, and cowpea chlorophyll mottle virus (CCMV), due to its homogeneous size and controllable assembly, is of great importance in the development of drug delivery vectors (Pille et al., 2021; Xue et al., 2023). L. X. Shen et al. engineered the capsid protein of CCMV using a modified block copolymer approach, a portion of the N-terminal structural domain of the CCMV capsid protein was replaced with a stimulus-responsive elastin-like polypeptide (ELP), and engineered virus-like NPs encapsulating DOX (EVLP@DOX) were then created. The ELP structural domain was transformed from a water-soluble state to a hydrophobic state, which stabilized the formation of the protein shell to stably encapsulate DOX, and the results showed that the EVLP@DOX NPs significantly promoted the anti-tumor immune response *in vivo* and demonstrated efficacy and safety in oncology therapy, compared to single DOX (Shen et al., 2024) (Figure 1).

Overexpressed DNA topoisomerase IIα (TOP 2A) is closely associated with the invasion and metastasis of breast cancers, and mitoxantrone (MTX) has been identified as a TOP-2A inhibitor. Conventional MTX drug delivery systems still suffer from low drug-carrying capacity and material carrier-related toxicity. S. S. Silva et al. constructed a carrier-free supramolecular nano assembly (QU-MTX-Fe) by coordinating Fe3+ ions with MTX and another TOP-2A inhibitor quercetin (QU) (Tang et al., 2020; Silva et al., 2021), in which the optimal dose ratio of MTX/QU was 1:2. PEG-modified QU-MTX-Fe(P- QU-MTX-Fe) exhibited good stability under physiological conditions and rapid release of the two drugs in an acidic TME. In a bilateral 4T1 breast cancer tumor model, *in-situ* injected P-QU-MTX-Fe could regress the growth of primary tumors and activate CD8-mediated anti-tumor immune responses. In conclusion, metal-iron coordination carrier-free supramolecular co-nano-assemblies of bi-DNA TOP-targeted inhibitors with a high drug-loading capacity are highly promising for clinical trials to achieve translation (Guan et al., 2022).

### 2.3 Plant alkaloids

Alkaloid drugs can inhibit mitosis or enzyme action, thus preventing protein synthesis essential for cell regeneration, which is an effective antitumor drug. Camptothecin (CPT) suffers from the drawbacks of low water solubility, poor structural stability, and toxic side effects, so it is necessary to continuously develop a series of corresponding structural modification strategies. M. T. Chen et al. based on host-guest recognition with CB [7] as the host and hydroxycamptothecin (HCPT) as the guest significantly increase the stability and solubility of CPT (Chen et al., 2022). Considering that CPT needs to exert its inhibitory activity in the nucleus, peptide amphiphiles can self-assemble into NPs under certain conditions and achieve subcellular deliver (Du et al., 2015). Q. X. Guo et al. used HCPT and β-CD which can be protonated at or below physiological pH to construct cationic nanomedicines. Like nuclear localization properties of cationic polymers, positively charged CD in acidic TME is prone to entry into the nucleus (Cortez et al., 2015). Moreover, since CD can deplete cellular ATP and reverse ATP-dependent drug efflux, enhancing cellular accumulation and anticancer activity of HCPT, HCPT-FFFK-CD amphiphiles were prepared and easily obtained HCPT-FFFK-CD hydrogels through a heating-cooling process, which significantly improved the cellular uptake of the drug, the nuclear accumulation (Guo Q. et al., 2021).

Glutathione (GSH) is one of the commonly used stimuli in the development of NPs. J. Zhan et al. reported an organelle-mimicking NP HCPT-peptide concatenation (HpYss), that self-assembly to nanofiber under sequential stimulation by extracellular alkaline phosphatase (ALP) and intracellular GSH. Such morphology transition mimics the dynamic cascade process of extracellular vesicles-mediated membrane transport and filament-mediated nuclear transport to deliver the drug to the nucleus, nucleus HpYss amplifies apoptosis and enhances the inhibitory effect of HCPT on tumor cells more than ten times (Zhan et al., 2021).

However, most morphologically transformable peptide-drug couplings (PDCs) as described above are dependent on physiological stimuli such as pH, enzymes, GSH, etc., which can to some extent differentiate cancer tissues from normal tissues, but the specificity is still poor, and PDCs responding to tumor metabolism-related biomarkers are important to precise tumor chemotherapy. Based on endogenous spermine (SPM) which is overproduced in breast, lung and CRC, C. Sun developed a novel supramolecular peptide-derived nanomedicine (FFVLK-CPT, PC) by non-covalent interactions among CB [7], peptide Phe-on-Phe-Val-Leu-Lys, and CPT, which self-assembled into amphiphilic NPs. In SPM overexpressing cancer cells, the host-guest pair could be competitively dissociated by spermine and release PC, which immediately formed a β-sheet structure, and subsequently reorganized into microfibrils, which dramatically improved the CPT in the tumor cells accumulation, retention, and sustained release, whereas this SPN exhibits considerably low toxicity to normal cells because of morphological stability and rapid exocytosis under low SPM condition (Sun et al., 2021b).

Vincristine belongs to the class of vinca alkaloids, which can disrupt mitosis by interfering with microtubule polymerization, thus inhibiting cell growth, and is one of the commonly used antitumor drugs in clinics, but the side effects are also obvious. S. Quader et al. developed extracellular PH-triggered NPs which can maintain a micellar structure with encapsulated drugs in circulation, and gradually release the drug in the tumor when correctly sensing the heterogeneous tumor PH. It was prepared by coupling deacetyl vincristine hydrazide (DAVBNH) with polymer micelles (PM) formed by PEG and poly (amino acid) (PAA) block copolymer. The NP could be stably bound within the micellar core during circulation under neutral pH conditions, and the drug release was activated once the PM reached the acidic TME and then proceeded continuously as the pH decayed both extracellularly and intracellularly. In glioblastoma xenografts and homozygous models, DAVBNH-loaded micelles increased the median survival rate to 1.4-fold and 2.6-fold, respectively, when compared with free DAVBNH (Quader et al., 2021).

Paclitaxel can terminate mitosis and cause tumor cell death by inhibiting microtubule depolymerization and is the only alkaloid that promotes microtubule formation while inhibiting microtubule protein depolymerization. C. Sun et al. used CB [7]-modified polylactic acid (CB [7]-PLA) and star-shaped polylactide (SSPLA) to form CB [7]- PLA/SSPLA NP (CS NP), and a new ROS-responsive polymer PEG–PBE–ADA (PPA) which prepared by phenylboronic ester (PBE) linked PEG and adenosine deaminase (ADA) was applied for non-covalent surface modification of CS NPs, finally form PPA-CS NPs. The NPs stay stable in circulation with lower immunogenicity and respond to ROS and GSH sequentially. ROS responsive de phosphorylation ensures efficient tumor accumulation and enhanced intracellular uptake, and GSH responsiveness leads to a specific release in cancer cells, exhibiting high anti-cancer bioactivity with low systemic toxicity (Sun et al., 2021a).

### 2.4 Other chemotherapeutic nanomedicines

Organometallic ruthenium (Ru)- arene complexes are promising anticancer agents, but their poor solubility in physiological media and lack of tumor uptake limit their further development. To address these challenges, D. Gopalakrishnan et al. developed aurum (Au) NPs coated with Ru (arene)-functionalized PNVP-Py. Specifically, RuII(η6-p-cymene) complex containing an NN bidentate ligand, one end could bind Ru complexes, and the other end could bind PNVP-Py on AuNPs, forming Ru (p-cym) (NN) (PNVP-Py)@AuNPs, showing better cytotoxicity against HT-29 CRC cells because of enhanced permeability to cell membranes. Nanoconjugate was found to induce apoptosis and inhibit expression of the cell cycle protein D1 which is required for cancer cell growth. It has been demonstrated that arginine deiminase (ADI) can cause tumor regression by amino acid deprivation therapy (AADT), and ADI can restore the sensitivity of cisplatin-18 or gemcitabine-resistant tumor cells. However, it suffers from inherent defects such as *in vitro* instability, low *in vivo* activity and reduced immunogenicity. X. Q. He et al. used β-CD derivatives hydroxypropyl-β-CD (HP-β-CD) and sulfobutylether -β-CD (SBCD) to encapsulate ADIs by noncovalent bond modification via molecular docking and form supramolecular complexes, respectively, which were then introduced into semi-permeable Within the biofilm, collectively referred to as ADI@MiSuNv, which incorporates an internal region of optimal enzymatic activity to facilitate the entry of substrate arginine into the microbial reactor, being converted to soluble citrulline and ammonia (He et al., 2021).

In addition to conventional chemotherapy, pressurized intraperitoneal aerosol chemotherapy (PIPAC) is considered a promising drug delivery method for the treatment of peritoneal metastases from unresectable ovarian cancer. H. Braet et al. investigated a pH-sensitive supramolecular polymer based on ureidopyrimidinone-PEG (UPy-PEG). When the acidity was reduced to pH 8.5 or less, a solution of UPy-PEG polymer was converted to a semi-solid hydrogel. Cisplatin-containing polyarginine hyaluronate NP (cis-Pt NP), albumin-conjugated paclitaxel (Abraxane), paclitaxel nanocrystals (PNCs), and DOX-loaded liposomes (DOX-LIP) were applied to test the UPy-PEG hydrogel’s ability to control both water-soluble (cisplatin, adriamycin) and insoluble (paclitaxel) drug substance release, with very promising results (Braet et al., 2023).

In summary, chemotherapy remains one of the main treatment options for cancer, and liposomal DOX (Doxil^®^) is the first anti-tumor nanomedicine approved by the FDA and was launched in 1995. Liposomal irinotecan, liposomal mitoxantrone, paclitaxel-albumin, etc. have been validated in clinical practice. However, due to factors such as inadequate drug design and individual differences, these already-used drugs still have many problems, such as generating new adverse reactions and drug-specific adverse immune reactions. The application of supramolecular nanomedicine in chemotherapy drugs effectively improves the targeting ability and drug utilization rate of chemotherapy drugs, greatly reduces systemic toxicity, and can also reduce cancer resistance (Table 1). In addition, supramolecular chemical drugs have the unique advantages of short development cycles and low development costs. While shortening the clinical application time of supramolecular chemical drugs, it ensures the economic efficiency of supramolecular chemical drug development and medical treatment. This will be beneficial for prolonging patient survival, alleviating economic pressure on patients, and improving their quality of life. Therefore, the research and improvement of supramolecular nanomedicines for chemotherapy drugs still need to be continued. However, the development potential of supramolecular nanomedicine for chemotherapy drugs is still more anticipated.

**TABLE 1:** Supramolecular nanomedicines based on chemotherapeutic drugs.

Carriers	Anti-cancer ingredients	Guidance /Stimulation	References
PEG-G4	CDDP	PH	Liu et al. (2021a)
TCPP、BA	Pt (II)	H2O2	He et al. (2024)
PAMAM-LA-CD, UCNPs, PEG-Azo	OXA-COOH, Pt (IV)	Azo reductase, pH	Yan et al. (2024)
CB [7]	DOX	TME	Chen et al. (2022)
PCL-MV, Nap-PEG), CB [8]	DOX	PH	Wu et al. (2021)
PCL-MV, Nap-PEG, (PSMA)-617, CB [8]	DOX	PH	Zhang et al. (2022a)
Pillar [5]arenes	DOX	PH/ROS, 660 nm light	Ding et al. (2022)
PCSTD-Gd	DOX	UTMD	Gong et al. (2023a)
5PHPMA35-b-PDPA75	DOX	PH	Albuquerque et al. (2021)
CCMV, EVLP	DOX	TME	Shen et al. (2024)
MTX/QU	MTX	PH	Guan et al. (2022)
CB [7]	HCPT	PH	Guo et al. (2021a)
GFFpY-ss-ERGD	HCPT	ALP, GSH	Zhan et al. (2021)
CB [7], Phe-on-Phe-Val-Leu-Lys	FFVLK-CPT	SPM	Sun et al. (2021b)
PEG-PAA	DAVBNH	PH	Quader et al. (2021)
CB [7]-PLA/SSPLA NP, PEG–PBE–ADA	PTX	ROS, GSH	Chowdhury et al. (2022)
SN38	PTX	PH, esterase	Li et al. (2021b)

Pt: platinum; DOX: doxorubicin; OXA: oxaliplatin; CDDP: cisplatin; HCPT: hydroxycamptothecin; DAVBNH: deacetyl vincristine hydrazide; UTMD: ultrasound-targeted microbubble destruction; SPM: spermine.

## 3 Supramolecular nanomedicines in immunotherapy

Cancer immunotherapy refers to a therapeutic method to remove and kill tumor cells by mobilizing the function of the body’s immune system and enhancing the body’s anti-tumor immune response, and the most clinically applied immune checkpoint inhibitors (ICI) of the programmed cell death protein 1 (PD1) and PD1-ligand (PD-L1) antibody class are currently used. However, due to various immune escape mechanisms, such as immunosuppressive TME, poor immunogenicity and insufficient cytotoxic T-lymphocyte (CTL) infiltration within the tumor, only about 20% of patients respond to immunotherapy (Liu and Sun, 2021). Therefore, the development of a novel and superior nanomedicine with the ability to sensitize tumors and reverse immunosuppression to enhance anti-tumor immune responses is imperative for tumor-amplifying immunotherapy.

### 3.1 Immune checkpoint inhibitor therapy

Cancer cells can inhibit the function of immune cells by expressing specific immune checkpoints that allow the cancer cells to evade surveillance and attack by the immune system. ICI enables the immune system to recognize and attack cancer cells by targeting these inhibitory molecules. Currently, anti-PD1 inhibitors inhibit the interaction of PD-1 and PD-L1, thereby maintaining T cell activation and stimulating anti-cancer immunity (Boussiotis, 2016), achieving important clinical progress in certain patients with advanced or metastatic tumors (Topalian et al., 2020).

**FIGURE 2:**
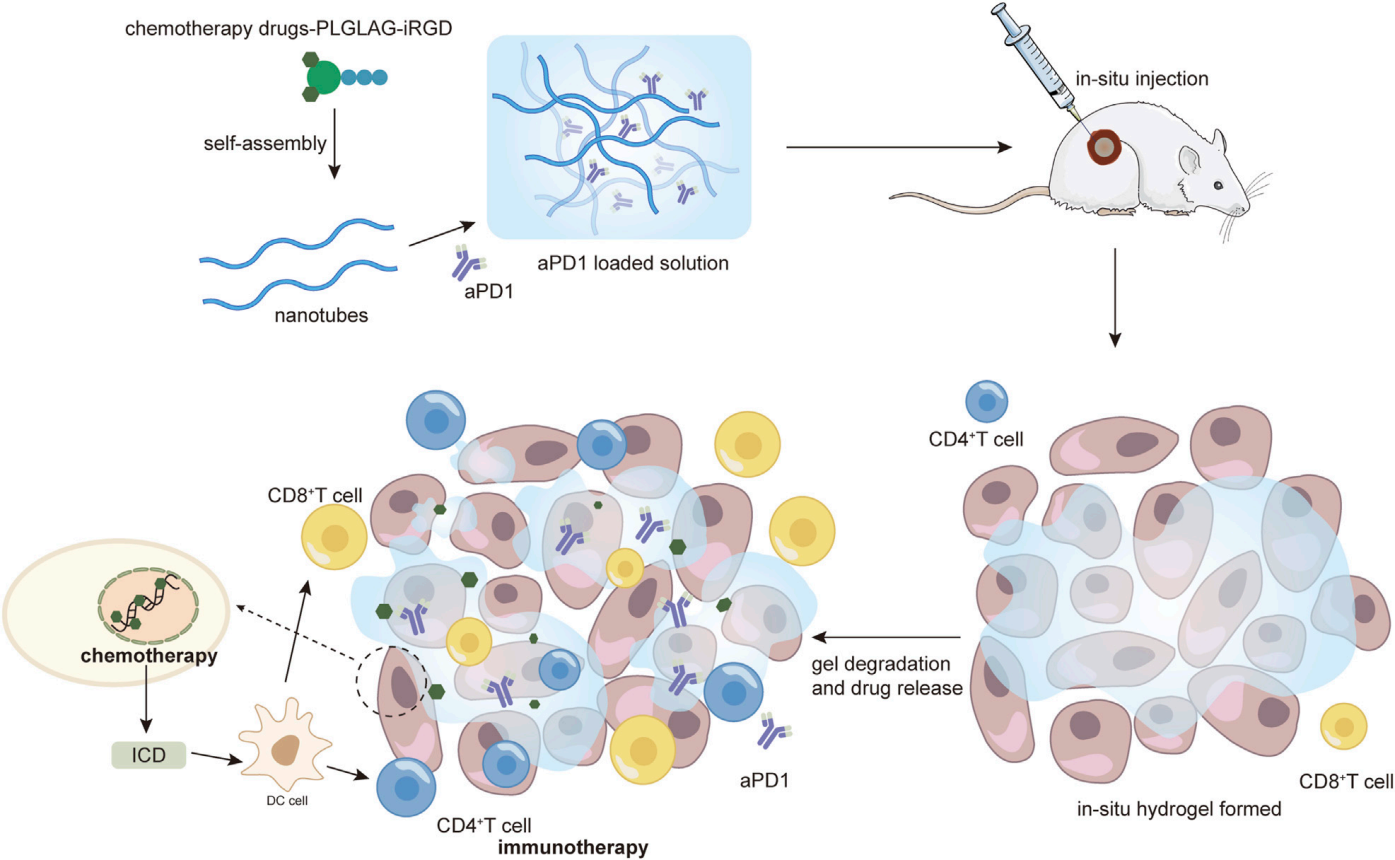
Demonstration of the enhanced immune-killing effect of nanomedicines by intratumoral injection of hydrogel as an example. From Wang et al. (2020b), reproduced with permission from AAAS.

IL15 and stimulator of interferon genes (STING) agonists have been reported to induce the expression of PD-L1 in tumors and PD1 on T cells. Specifically, IL15 selectively stimulates memory CD8 T cells and has been shown to enhance cell-mediated immunity, resulting in anti-tumor effects (Mathios et al., 2016). STING agonists like c-di-AMP (CDA), are another emerging class of immunostimulatory factors, whose activation triggers the stimulation of type I interferon production by antigen-presenting cells thereby promoting the activation of tumor-specific T cell responses (Sun et al., 2013). F. H. Wang et al. developed a supramolecular hydrogel for delivery of aPD1, IL15, and CDA-S formed by self-assembly of peptide-based building blocks, consisting of the peptide-based amphiphilic molecule DOCA-PLGLAG-iRGD. The *in-situ* injection of DOCA-PLGLAG-iRGD supramolecular filaments (SFs) solution induced the formation of molecular hydrogels, prolonging intra-tumor retention and facilitating tumor penetration of immunotherapeutic agents. Matrix metalloproteinases (MMPs)-2 are a group of enzyme associated with cancer progression that is overexpressed in a wide range of malignant tumors (Wang F. et al., 2020). PLGLAG peptide is cleavable by MMP-2, causing degradation of SFs and enhancing the release of aPD1 (Figure 2). The experimental results showed that both aPD1/IL15-SF and aPD1/CDA-SF combination therapy significantly increased T-cell infiltration and attenuated the immunosuppressive response, thereby overcoming adaptive immune resistance induced by IL15-SF or CDA-SF monotherapy resulting in complete regression of established large tumors with a survival rate of 100%. And more importantly, the topical hydrogel system triggered long-lasting T-cell memory and systemic anti-tumor immunity, suggesting that this strategy has excellent potential for preventing tumor recurrence and metastasis (Wang F. et al., 2023).

Indoleamine 2,3-dioxygenase (IDO) inhibitor-mediated immune checkpoint blockade therapies can also be used to reverse immunosuppression (Guo Y. et al., 2021). IDO is a key negative feedback enzyme in immunosuppressive TME formation (Campesato et al., 2020), which exerts its immunosuppressive function by facilitating the enzymatic process of conversion of L-tryptophan (Trp) to L-kynurenine (Kyn), which accumulates to provide the necessary nutrients for rapid tumor growth, and the degradation of the Trp interferes with the survival and activity of CTLs (Su et al., 2018). NLG919, an IDO inhibitor used in phase II clinical trials, stimulates positive feedback in the immune system by modulating the Trp-Kyn pathway (Ding et al., 2021; Liu L. et al., 2023). Pyroptosis is known as inflammation-associated programmed cell death, and tumor cells undergoing pyroptosis can induce immunogenic cell death (ICD) by releasing a set of molecules known as danger-associated molecular patterns (DAMP), which are important immunologic adjuvants for the recruitment and maturation of antigen-presenting cells, stimulation of pre-existing anticancer immune responses, and enhancement of the anticancer efficacy. Promoting cellular pyroptosis is therefore an important approach for cancer immunotherapy, and previous studies have shown that nitric oxide (NO) can induce macrophage pyroptosis, but NO-based gas therapy has not been reported to trigger cancer cellular pyroptosis.

Based on the role of pyroptosis and IDO in tumor immune response, researchers hypothesized that the combination of NO-mediated pyrokinesis inducers with IDO inhibition-based immunotherapy is a promising approach for the construction of novel nanomedicines. W. T. Hu et al. use a CD-based supramolecular self-assembly system to prepare this nanomedicine. NLG and NO donors were conjugated with CD via a thioketal (TK) moiety as the ROS-trigger linker and an S-nitrosothiol bond (-SNO) as the GSH-trigger linker, forming NLG-CD-SNAP. NLG-CD-SNAP can be synthesized via subject-guest recognition and hydrophobic interactions to self-assemble into polymeric nanodrugs (NCSNP). Upon entry into the TME, NLG and NO are sequentially excreted via GSH/ROS-mediated catabolism; NO release causes pyroptosis, which can release immunostimulatory cytokines and restore strong inflammatory effects, while NLG is activated to cut off the IDO pathway, which can reverse the immunosuppressive environment, simultaneously triggering pyroptosis and impeding IDO-associated immune escape for self-cascade amplification of antitumor immunotherapy, which will pave a new path for cancer immunotherapy (Hu et al., 2024).

### 3.2 Tumor vaccines

Poor immune response activation has become a major obstacle to the successful application of ICI therapy in most tumor types. Effective immune activation and maintenance of CTL cell populations are essential prerequisites for the immune effector process. Tumor vaccine is an immune intervention strategy that uses tumor-specific antigens or tumor-associated antigens to activate body-specific immune responses to kill tumor cells and prevent tumor recurrence and metastasis by establishing long-term anti-tumor immune memory. NPs loaded with cytotoxic agents and immune agonists can be used as *in situ* cancer vaccines to stimulate tumor-specific immune responses using the tumor itself as the source of antigen (Patel et al., 2019). However, there is a contradiction in using a single type of NPs to meet all the requirements in the series of immune activation processes *in vivo*, including antigen release, antigen capture, antigen-presenting cell activation, antigen presentation, and T-cell initiation. e.g., cationic NPs, which are prone to capture antigens, perform poorly in circulation (Di et al., 2021), while large NPs that tend to accumulate in tumor tissues show low efficiency in the accumulation of tumor-draining lymph nodes (TdLNs) (Schudel et al., 2019). Therefore, environmentally responsive nanomaterials also play a significant role in tumor vaccine development.

Y. Zhang et al. prepared a supramolecular assembled programmable immune activation nanomedicine (PIAN) as an *in situ* vaccine for cancer utilizing poly-[(N-2-hydroxyethyl)-aspartamide]-Pt (IV)/β-CD (PPCD), CpG/PAMAM-TK-adamantane (CpG/PAMAM-TK-Ad), and methoxy-PEG-thioketal-adamantane (mPEG-TK-Ad). The optimized PIAN leads to multiple consecutive steps after being injected into the body: 1) circulation in the bloodstream, 2) accumulation in tumor tissues due to enhanced permeability and retention effects, 3) CpG/PAMAM and PEG segregate in response to high ROS levels within the tumor tissue, 4) endocytosis of PPCD by tumor cells, which promotes tumor cell death and antigen release, 5) capture of released antigen by CpG/PAMAM, and 6) return of tumor antigen-specific effector T-cells to the tumor bed to exert anti-tumor effects. In conclusion, this supramolecular tumor vaccine stimulates multiple steps of the immune activation process and triggers a potent antitumor immune response, and synergistically inhibits tumor growth with αPD-L1 (Zhang Y. et al., 2021).

As indispensable components of tumor vaccines, immune adjuvants can significantly increase the magnitude, breadth, and durability of anti-tumor immunity (Kim et al., 2015). However, immune adjuvants currently used as tumor vaccine-based immunotherapy suffer from weak immunogenicity, insufficient cellular internalization, short half-life, and a single mode of action (Chen et al., 2019; Liang et al., 2021; Zhang L.-X. et al., 2021). Therefore, there is an urgent need to design and construct novel immune adjuvants with multiple biological activities for cascade vaccination of tumor vaccines. Evocation of ICD from tumor cells is significantly beneficial for amplifying specific anti-tumor immunity of tumor vaccines. However, most of the ICD present is associated with immune-tolerant apoptosis, in which apoptotic cells usually remain immune-silenced (Su et al., 2022). Unlike apoptosis, ferroptosis and necroptosis are two types of programmed cell death with high immunogenicity. The former causes ICD by releasing DAMPs (Yang et al., 2021) and the latter causes the release of cellular contents such as immunogenic DAMPs to trigger ICD in tumor cells (Vanden Berghe et al., 2014). Therefore, exploring immune adjuvants with the ability to induce ICD in tumor cells undergoing immunogenic necroptosis and ferroptosis could largely enhance antitumor immunity. Based on this, W. J. Feng et al. constructed Fe(III)-Shikonin network (FeShik) nanomedicines as multifunctional immune adjuvants for ICD stimulation and tumor vaccination. After being phagocytosed by tumor cells, FeShik nanomedicine breaks down into Fe2+ and shikonin in response to the TME, leading to ICD of tumor cells by inducing ferroptosis and necroptosis, and ICD-released tumor cell lysates and pro-inflammatory cytokines not only stimulate DC maturation and antigen cross-presentation, but also promote macrophage repolarization and CTL infiltration, thus activating adaptive immune responses against solid tumors. Nanovaccines (TF@FeShik) prepared with loaded tumor cell fragments (TFs) exhibit potent antitumor effects for eradication of primary tumors, strong inhibition of distant tumor growth, and long term immune memories for tumor metastasis and recurrence (Feng et al., 2023).

Currently, peptide-based tumor vaccines are widely used in cancer immunotherapy due to their high degree of personalization, low cost, ease of manufacture, and favorable safety profile (Liu W. et al., 2021). However, compared with conventional vaccine strains involving subunit proteins and inactivated viruses, their antitumor efficacy is limited due to insufficient antigen-specific CD8 T cell responses. The underlying reason for this is the heterogeneity of tumor antigen expression, where tumor cells that do not express the targeted antigen can evade immune recognition and elimination by antigen-specific CD8 T lymphocytes, leading to immunotherapy failure. Therefore, the establishment of a tumor vaccine such as one that delivers multiple peptide antigens simultaneously seems to solve the problem to some extent. Inspired by the naturally occurring assembly motifs in proteins, self-assembling peptide hydrogels using amino acids as their building blocks have been developed as promising vaccine scaffolds (Li S. et al., 2021; Liu et al., 2022). H. J. Song et al. chose the nonimmunogenic self-assembling peptide FEFEFKFK as a carrier for antigenic epitope, coupled with several representative antigens that have been reported in tumor vaccine clinical studies such as glycoprotein 100,209–217 (gp100209-217), tyrosinase 369-377 (Tyr369-377), and melanoma antigens recognized by T-cells 1 (MART-126-35). The expression of the selected tumor antigens on the surface of B16 cells was detected by flow cytometry and a trivalent peptide hydrogel vaccine was designed according to the ratio between the three antigens, and the vaccine could improve antigen presentation to DCs due to its fibrous structure and induce inflammatory responses, it was meanwhile immunological adjuvant itself. Ultimately, it greatly facilitated antigen presentation to the DC and its subsequent homing to the dLN and triggered a broad-spectrum anti-tumor CD8 T-cell response, which resulted in significant inhibition of B16 tumor growth, but not all tumors were completely eradicated in each treatment group, suggesting that further optimization of vaccine dosage is required to enhance the immune response of cytotoxic CD8 T-cells in future work and that the assembly of each epitope was also adjusted ratios to tailor personalized peptide vaccines. Moreover, incorporating ICI to enhance the efficacy of peptide hydrogel-based immunotherapy to eradicate established tumors (Song et al., 2023).

### 3.3 Other

In addition to immune checkpoint inhibitor therapies and tumor vaccines, immunomodulators play an important role in the treatment of malignant tumors. Cancer immunity is closely related to the immunosuppressive TME induced by tumor-associated macrophages (TAMs) and regulatory T cells (Tregs) (Shamdani et al., 2020), notably, M2 -like TAMs are prevalent in tumor-infiltrating immune cells and their high abundance is usually associated with poor prognosis. Because of the obvious side effects of direct elimination or depletion of TAMs, the possibility of reprogramming pro-tumorigenic M2 macrophages into anti-tumorigenic M1 macrophages was considered. Resiquimod (R848) can polarize TAM from an M2-like phenotype to an M1-like phenotype (Jiang M. et al., 2021), however, the pharmacokinetics of R848 are not optimal, especially when administered in the peritoneal cavity; due to its relatively small size, R848 rapidly diffuses and causes subsequent systemic toxicity (Varshney et al., 2021). In contrast, the effectiveness of TLR7/8 agonists in cancer therapy can be improved by employing a drug delivery strategy that enhances drug retention and targeted delivery while minimizing off-target toxicity (Lan et al., 2020). Yuan H et al. used poly (lactic-co-glycolic acid) (PLGA) nanocarriers to encapsulate the R848 (R848@NPs), and 2-HP-β-CD to optimize the PLGA carriers (CD@R848@NP), and this modification improved drug solubility and macrophage polarization while protecting the NPs from *in vivo* phagocytosis (Yuan et al., 2024). In addition, H. J. Jiang et al. designed and prepared supramolecular chiral polymeric micelles (SCPM) by complexing the tumor-associated antigen ovalbumin (OVA) with chiral basic histidine-modified poly (ethylenimine) (PEI), and the results showed that the introduction of chirality could simultaneously reduce the toxicity of PEI and promote cellular uptake as well as antigen processing and presentation. Moreover, D-chirality increased the highest expression level of co-stimulatory molecules on DCs compared to the corresponding L-chirality and achirality, and the interaction between the chirality of the synthesized material and the immune system inspired alternative ideas for the development of immunotherapy (Jiang et al., 2023).

In conclusion, the application of supramolecular nanotechnology in immunotherapy is currently limited and still needs to be further explored in specific targeting of immune checkpoint inhibitors, antigen delivery in tumor vaccines, immune adjuvants, and even more.

## 4 Supramolecular nanomedicines in targeted therapy

In addition to traditional therapies such as chemotherapy and radiotherapy, targeted therapy, which utilizes specific molecular targets to selectively act on cancer cells, plays an important role in the treatment of malignant tumors due to its advantages of good efficacy and low side effects.

### 4.1 P53 pathway

P53, as a tumor suppressor protein, inhibits p53 ubiquitylation when cells are subjected to DNA damage, oxidative stress, etc., resulting in a rapid increase of intracellular p53 protein level, triggering cell cycle arrest or apoptosis. Through such a classical p53 signaling pathway, damaged cells can be removed *in vivo* via the apoptotic pathway, thus preventing the accumulation of potential tumor cells, and the P53 pathway is a potential target. The main approach is to target the negative regulator of p53, mouse double minute 2/X (MDM2/MDMX). Using small molecule compounds, which can effectively dissociate the p53/MDM2 complex and inhibit MDM2 mediated p53 ubiquitination and degradation, inducing the activation and accumulation of wild type p53 in tumor cells.

Self-assembled D-peptide nanomedicines have attracted increasing attention due to their low toxicity, good biocompatibility, high specificity and affinity for biomolecular targets (Sato et al., 2018), but their usual impermeability to cell membranes limits their action on intracellular targets (Jeena et al., 2020). Previous studies have shown that the cell-penetrating ability of peptides is dependent on the regulation of neuropilin-1 (NRP1), a protein that is overexpressed in cancer cell membranes and that NRP1-targeted peptides can efficiently penetrate deeper into tumor tissues and enter cancer cells (Detroja et al., 2017). Y. K. Zhou et al. successfully identified a self-assembled D-peptide supramolecular nanomedicine (NMTP-5) targeting NRP1 and MDM2 using a structure-based virtual screening technique, which self-assembled into nanofibers in an aqueous solution, and after entering the cytoplasm of cancer cells by targeting NRP1, NMTP-5 activated the p53 signaling and induced cell death *in vivo* by interfering with the MDM2-p53 interaction. *In vivo* and *in vitro* experiments showed that NMTP-5 has strong biostability, cellular uptake properties and anticancer efficacy (Zhou et al., 2021).

**FIGURE 3:**
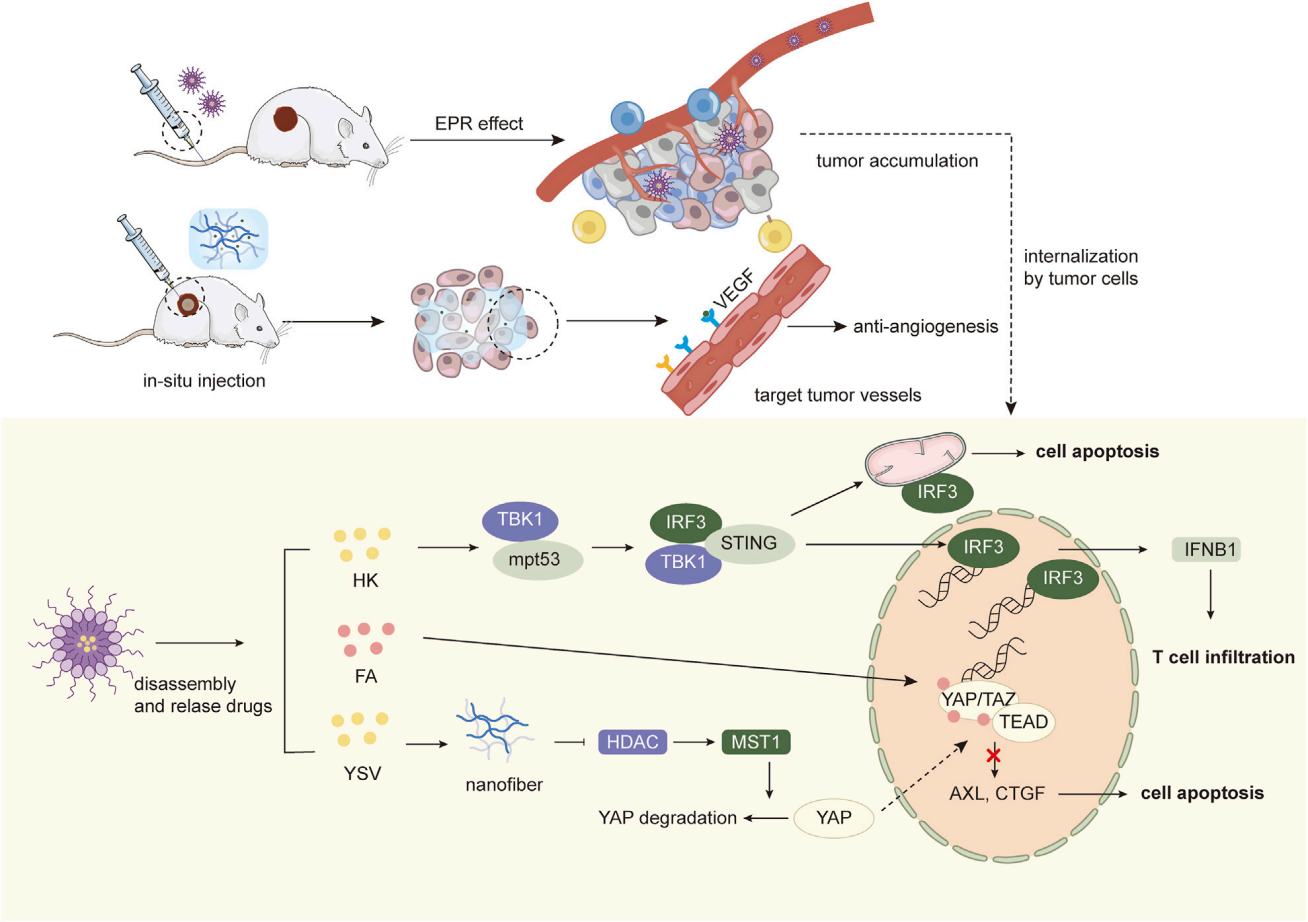
Schematic illustration of targeted therapy combined with nanomedicines.

In addition, H. Ji et al. investigated the preparation of self-assembled NPs targeting p53 mutant tumors using honokiol (HK). First, HK formed self-assemblies controlled by interacting hydrogen bonds in aqueous solution and aggregated at the tumor site via EPR-mediated passive targeting. After endocytosis by tumor cells, the inter-bound HK molecules were hydrolyzed in an acidic environment and released, generating sustained HK levels in tumor cells, which play a role in inhibiting cancer cell proliferation and inducing apoptosis by activating BMP7 and up-regulating TGF-β1 expression, activating p53 expression through the non-classical TGF-β1 signaling pathway. HK NPs-mediated downregulation of mutp53 significantly increased the synergistic anticancer effects of cisplatin, adriamycin and 5-Fu in HT-29 cells. Meanwhile, mutp53 downregulation could restore TBK1 activity, inducing transcription of IRF3 target genes and IRF3-dependent apoptosis. Moreover, NPs-induced unbinding and degradation of the mutp53/TBK1 complex switched the TME from cold to hot, which restored the immune system to effectively suppress tumor growth (Ji et al., 2022) (Figure 3).

M. R. Schnorenberg elucidated synergistic treatment in diffuse large B-cell lymphoma (DLBCL) by triggering apoptosis by reactivating the tumor protein p53 with the suture peptide ATSP-7041, and BH3 mimetic ABT-263 (navitoclax) was administered to enhance sensitivity to BCL-2 family. While this combination was highly effective in activating apoptosis in DLBCL *in vitro*, it was highly toxic *in vivo*, resulting in a very narrow therapeutic window. Therefore, a CD19-targeting polymer set was prepared using a block copolymer of PEG disulfide-linked to poly (propyl sulfide) (PEG-SS-PPS) to delivery ATSP-7041 into DLBCL cells. Through packaging and targeted delivery of the suture peptide, the combination maintains therapeutic potency while minimizing its toxicity (Schnorenberg et al., 2023).

### 4.2 Hippo pathway

The Hippo pathway is a growth inhibitory kinase cascade that can participate in tumorigenesis by manipulating cell proliferation, division and death. MST1/2 (Hippo kinase) and MAP4K1-7 (Hippo-like kinase) phosphorylate and activate LATS1/2, which phosphorylates YAP and TAZ, and the activated YAP or TAZ proteins translocate to the nucleus, where they physically interact with transcriptional enhanced associate domain (TEAD) to regulate expression of genes that control cell proliferation and apoptosis. Gene expression that controls cell proliferation and apoptosis. Currently, the screening of Hippo pathway-targeting drugs mainly focuses on how to inhibit the protein-protein interactions between YAP/TAZ and TEAD, and previous studies have shown that the small-molecule drug flufenamic acid (FA) can target the palmitoylated pocket of TEAD, thereby affecting its folding, stability, and binding to YAP (Bum-Erdene et al., 2019). In addition, histone deacetylase inhibitors (HDACi) can upregulate MST1 activity and inhibit YAP expression. Therefore, Z. L. Wang et al. designed a supramolecular NPs consisting of hydrophobic peptide fragments modified with FA and YSV, hydrophilic PEG fragments with double reactive sites of ester and disulfide bonds, which could be assembled into “core-shell” shaped nanorods *in vitro* and could be applied to tumors via EPR effect of PEG. After being endocytosed by tumor cells, they form peptide nanofibers *in situ* under the dual stimulation of overexpressed GSH and esterase and release small molecule targeting drugs FA. Active nanofibers containing YSV promote the upregulation of the upstream MST1 kinase through the inhibition of HDAC, thus causing YAP phosphorylation and cytoplasmic degradation. At the same time, free FA released *in situ* enters the nucleus and binds to TEAD, which acts synergistically with the Hippo pathway to maximize the blockade of YAP-TEAD interactions, thereby inhibiting the transcription of growth factor genes (e.g., CTGF, CYR61, and AxL) to exert antitumor effects. Furthermore, activation of the Hippo pathway blocks the cell cycle, showing excellent radio-sensitizing effects on cancer cells, thus producing synergistic anti-tumor effects with radiation therapy and prolonging survival (Wang et al., 2022).

### 4.3 Anti-angiogenesis targets

In addition to targeting tumor cells, anti-angiogenic therapies targeting inhibition of the vascular endothelial growth factor (VEGF) pathway have shown clinical benefit in vascular-rich tumors, such as HCC. To modulate cell types in TME and improve the therapeutic efficiency of anti-angiogenic therapy, X. Liu et al. developed a supramolecular hydrogel drug delivery system (PLDX-PMI) by assembling anti-angiogenic nanomedicines (PCN-Len NPs) and oxidized dextran (DX), loading with TAMs-reprogramming polyTLR7/8a nano regulators (p (Man-IMDQ) NRs), which are capable of localized sustained release of nanodrugs and nano modulators in response to the acidic environment of tumors for *in situ* cancer therapy. The nanomedicine PCN-Len NPs loaded with lenvatinib target tyrosine kinases in vascular endothelial cells to downregulate the expression of VEGF-A and Ang-2 and reduce micro vessel density, and the p (Man-IMDQ) NRs reduce pro-angiogenic activity through mannose receptor by repolarize pro-angiogenic M2 TAMs to anti-angiogenic M1, further reducing VEGF secretion, impeding the migration and proliferation of vascular endothelial cells, enhancing the anti-tumor angiogenic effect of lenvatinib (Liu X. et al., 2023).

### 4.4 Other targets

Exportin 1 (XPO1) is a transporter protein whose upregulation leads to the inactivation of tumor suppressors and loss of anti-tumor function in many cancers, making it a validated drug target [13]. Ataxia telangiectasia and Rad3-related (ATR) are key regulators of the DNA replication stress response and the activation of the DNA damage activation checkpoint, and previous studies have shown that XPO1 inhibition in combination with ATR inhibition may be one of the therapeutic approaches for malignant tumors. Therefore, L. Gong et al. constructed Au-based supramolecular NPs (AA@G) by combining ATR small molecule inhibitor AZD6738 and XPO1 peptide inhibitor AP using human serum albumin, an *in vitro* and *in vivo* experiments demonstrated that AA@G had extraordinary biocompatibility and enhanced therapeutic efficacy by inducing HCC cell cycle blockade, facilitating DNA damage and inhibiting DNA repair (Gong L. et al., 2023).

Like immunotherapy, supramolecular nanomedicines based on targeted therapies are still mainly in the preclinical exploratory stage, and more evidence is still needed on how supramolecular nano designs can further improve the efficacy and safety of targeted drugs due to their unique targeting effects.

## 5 Supramolecular nanomedicines in photochemical therapy

Photochemical therapy is a rapidly developing disease treatment modality, mainly including PTT and PDT, which utilizes different wavelengths of light to induce photothermal or photochemical changes in target tissues, raising local temperatures or generating cytotoxic ROS to kill tumor cells (Li et al., 2020). Photothermal transforming agents (PTAs), and photosensitizers (PSs) are the core and foundation of PTT and PDT. Supramolecular nanomedicines have an important place in PTT and PDT due to their specific targeting and multiple stimulus responsiveness (Table 2).

**TABLE 2:** Supramolecular nanomedicines based on photochemical therapy.

Photothermal agent	Carrier or others	Principle	Light wavelength (nm)	Therapeutic effects	References
Au-Fc NPs	Fe_3_O_4_-CB [7]-NPs	host-guest interaction	808	PTT + CT imaging	Cheng et al. (2021)
CuS NP	β-CD-Fc	host-guest interaction	808	PTT + CT imaging	Li et al. (2021a)
IR1064、Gd		Metal coordination	1,064	PTT + PAI + MRI	Lei et al. (2022)
Cy7-TCF	OTP	Self-assembly	808	PTT	Lin et al. (2022)
Cy7-TCF	IMC	Self-assembly	808	PTT	Mu et al. (2021) Ma et al. (2023)
IR780	SAC4A	host-guest interaction	808	PTT	Zhang et al. (2022b)
IR817	BSA	Hydrophobic interaction	808	PTT	Wang et al. (2023a)
cationic porphyrin	ATP	Electrostatic interaction	635	PDT	Li et al. (2021c)
Por (PEG)4		Hydrophobic interactions and π - π interactions	675	PDT	Casellas et al. (2022)
Gd–TCPP	PEI and PGA-g-mPEG	Noncovalent electrostatic interactions	635	PDT	Chen et al. (2021)
Hyp	MnO_x_	Metal coordination	980	PDT	Zhao et al. (2023)
CyOA NP	oleic acid	Self-assembly	660	PDT	Wang et al. (2023b)
DTTP	Pt (PEt3)2(OTf)2	Coordination effect	808	PTT + PDT	Qin et al. (2022)
RuDA	PEO-PPO-PEO	Coordination effect	808	PTT + PDT	Xu et al. (2022a)
B4		Self-assembly	808	PTT + PDT	Cheng et al. (2022)

PTT: photothermal therapy; PDT: photodynamic therapy; PAI: photoacoustic imaging; MRI: magnetic resonance imaging.

### 5.1 Photothermal therapy

PTT is a medical treatment technique that uses light energy to generate localized heat for therapeutic purposes. This heat is produced by photosensitive agents, such as nanoparticles or dyes, that absorb specific wavelengths of light and convert it into heat so that high temperature induces apoptosis of the tumor cells. However, low photothermal conversion efficiency is the main obstruction, and improving the conversion efficiency by improving the structure of PTA is one method. Supramolecular photothermal nanomedicines constructed by self-assembly of biomolecules are promising drug candidates.

#### 5.1.1 Precious metal-based supramolecular nanomedicines

Inorganic NPs have attracted much attention in the biomedical field due to their excellent biocompatibility and tunable physical, chemical, and biological properties, but these properties are usually dependent on their size, morphology, and composition. Small NPs are highly tissue-penetrating and have the potential to reach deep solid tumor tissues (Zhao et al., 2019) but they are also prone to be removed too quickly (Yang et al., 2019). Therefore, Targeted accumulation and *in situ* aggregation of small NPs to form larger structural treatments is a rational design for nanomaterials.

Au NPs can be used for tumor-specific PTT because of their optical property of localized surface plasmon resonance (LSPR), so they can efficiently convert NIR into heat. However, various stimuli-responsive ligands currently available to drive the aggregation of Au NPs are characterized by difficulties in preparation, small application range, poor targeting, etc. (Hong et al., 2020). To improve this series of problems, Q. Cheng et al. anchored CB [7]-capped Fe_3_O_4_ NPs (Fe3O4-CB-NPs) in tumor tissues to serve as an artificial target, and ferrocene (Fc)-capped Au NPs (Au-Fc NPs) could form an *in-situ* aggregation within tumor tissues through a strong, multi-point CB [7]-Fc host-guest interaction, realizing efficient accumulation of Au NPs within the tumor for PTT and locally enhanced cancer CT imaging. Animal experiments revealed that the tumor size was reduced by 63% after PTT (Cheng et al., 2021).

Small copper sulfide NPs (CuS NPs) have also been widely investigated due to their high photothermal conversion efficiency, low cost, and ease of preparation and functionalization (Yang et al., 2020). However, CuS NPs are usually captured by the reticuloendothelial system (RES) or rapidly cleared through the kidney, resulting in insufficient accumulation in tumors (Bourquin et al., 2018). The inflammatory nature of tumor tissues suggests that immune cells can be used for tumor-specific drug/nanodrug delivery along with internalized drugs, especially macrophages that are highly phagocytic and tolerant to drugs/NPs and plastic in their response to tumors (Xia et al., 2020). J. Y. Li et al. developed a novel macrophage hitchhiking delivery system based on supramolecular self-aggregating nanomedicines in macrophages. Firstly, macrophages sequentially phagocytose β-CD-modified CuS (CD-CuS) and Fc-modified CuS (Fc-CuS), forming a large supramolecular aggregate through multiple strong β-CD-Fc host-client interactions, hereby inhibiting their efflux from macrophages. After 2 h of intravenous injection of CuS internalized macrophage, a gradual increase in CT signal in the tumor was found, indicating that macrophages can transport CuS to the tumor site. Oxidation of Fc in an inflammatory tumor environment leads to the decomposition of aggregates and the release of small-sized CuS NPs, enhancing the PTT effect. It was found that 4 h after injection and 5 min after placing the tumor under laser irradiation, only the temperature of the tumor site increased significantly, and the effect on the surrounding healthy tissues was very small, whereas the control group caused almost no damage to the tumors. In conclusion, the researchers have used supramolecular chemistry to provide a novel cell-hitchhiking drug delivery strategy, which not only minimizes the premature leakage of nanomedicines but also greatly improves the drug delivery efficiency and tumor penetration ability, leading to safe and effective tumor therapy (Li J. et al., 2021).

#### 5.1.2 NIR dyes-based supramolecular nanomedicines

NIR dyes are a class of compounds with photon absorbing and emitting properties in the NIR spectral range, and the metal supramolecular nano-assemblies (MSNAs) formed by coordination of dye molecules with metal ions not only retained the intrinsic optical properties of the dye molecules but also exhibited enhanced photothermal conversion ability and photodynamic activity. However, the current dye molecule-based MSNAs can only operate within the first near-infrared (NIR-I, 650–950 nm) biological window, but the second NIR biological window (NIR-II, 950–1700 nm) offers deeper tissue penetration and higher maximum allowable exposures to NIR laser light (Jiang Y. et al., 2021; Xiang et al., 2021). Therefore, the development of NIR-II dyes MSNAs with efficient absorption and good photostability is important for NIR-II phototherapy. S. Lei et al. found gadolinium-based metal dye supramolecular nanocomponents (Gd@IR1064) with the intrinsic optical properties of NIR-II essential dye, which not only exhibit NIR-II photoacoustic, fluorescent, and magnetic resonance imaging capabilities to penetrate into the deep tissues but also have enhanced photothermal conversion properties to induce thermotherapy, achieving remarkable tumor elimination results (Lei et al., 2022). Moreover, nanostructured small molecule PTAs are “passively” targeted to tumors based on EPR effects, but according to meta-analysis studies, these NPs accumulate in less than 1% of tumors even in high EPR xenografts due to the lack of specific targeting ligands (Liu et al., 2019), thus giving PTA higher targeting is important for improving PTT efficacy.

P. Lin et al. used osteosarcoma (OS) as a model of heterogeneous cancer and screened OS-tailored targeting peptide (OTP, sequence TPPRVPLLTFGS) by phage display technology. OTP was then coupled with 2D nanodiscs of heptamethine cyanines (Cy7) which are synthesized using tricyanofuran (TCF) as the acceptor and benzothiazole (BTH)/indole (IND) as the donor, the dye self-assembled to form stable nanostructures, showing high photothermal conversion efficiency and excellent passive tumor targeting ability (Mu et al., 2020). After modifying its surface with tumor-specific targeting peptides, such tumor-targeting nanodiscs (T-ND) could successfully bind to certain receptors on heterogeneous tumor cells and exhibit specific affinity for OS cells and tissues. The experimental results showed that after a single dose of intravenous injection into tumor-bearing mice, T-ND can not only act as a PTA to precisely destroy tumors and inhibit tumor growth, but also effectively penetrate deeply into tumor tissues with ultra-long tumor retention up to 24 days, which is a 7-fold increase compared to ordinary nanomedicines (Lin et al., 2022).

Cyclooxygenase-2 (COX-2) is an attractive target in tumor targeting because it is absent or lowly expressed in most normal cells but significantly upregulated in many malignant tumors (Dannenberg et al., 2005). Indomethacin (IMC), a COX-2-targeting ligand, is linked to Cy7-TCF to form a small-molecule-based PTA (Cy7-TCF-IMC), which can be self-assembled into nanosheets in aqueous solution for passive targeting to the tumor site via the EPR effect, providing a chance for COX-2 targeting system. *In vitro* cellular experiments demonstrated that Cy7-TCF-IMC shells exhibited excellent selectivity for COX-2 overexpressing HeLa cells, and *in vivo* photoacoustic imaging confirmed that the shell exhibited effective targeting of COX-2-positive tumors (Mu et al., 2021). In addition, H. H. Ma et al. designed and synthesized a ROS-based PS (Cy7-TCF-TK-IMC) by coupling Cy7-TCF to IMC via a thioketa (TK) linker, which then self-assembled into NPs and accumulate in tumors via the EPR effect, then actively targeting tumor cells via specific IMC/COX -2 pattern. Unlike Cy7-TCF-IMC studied by P. Lin et al., the overproduced ROS in cancer cells cleaves the TK moiety and IMC is removed from the NPs, followed by the release of small-sized Cy7-TCF-SH NPs to improve tumor penetration. Cy7- TCF-TK-IMC NPs can be activated by the ROS threshold to enhance photothermal conversion in tumor cells, but not to achieve higher thermotherapy in normal cells, which can overcome the photothermal toxicity to adjacent healthy tissues (Ma et al., 2023).

IR780 is a lipophilic small molecule anthocyanin dye with maximum absorption at 780 nm, which has good photothermal conversion performance, stability, and fluorescence imaging properties, and has attracted wide attention in tumor phototherapy. However, IR780 is insoluble in water, rapidly metabolized and toxic, limiting its further development and utilization. SAC4A is a hypoxia-responsive macrocyclic host that exhibits strong binding affinity for various anticancer drugs under normoxia conditions and degrades under hypoxic conditions to amino-calix [4]pyrenes (NH2C4A) with low affinity for drugs (Chen et al., 2015). The synthesized supramolecular formulations of SAC4A and IR780 (IR780@SAC4A) have good water solubility, and when IR780@SAC4A reaches the tumor site with high expression of hypoxic-induced reductase (Ma et al., 2015), the azo linker of SAC4A could be reduced by reductase to form NH2C4A, leading to the hypoxia-responsive release of IR780. In addition, both SAC4A and NH2C4A could improve the photostability and photothermal conversion of IR780, thus further enhancing the efficacy of PTT (Zhang T.-X. et al., 2022); IR-817 is a new near-infrared fluorescent dye synthesized by coupling IR-808 with dicyclohexylcarbodiimide, which is also still plagued by poor water solubility, aggregation-caused quenching (ACQ) effect, photobleaching, short imaging time, and toxicity, etc. It has been shown that certain hydrophobic regions of albumin can bind to certain cyanine dyes to form albumin@dye complexes, which improves the dyes’ biocompatibility and photostability, while albumin encapsulation will produce NPs with relatively large size and long half-life in blood, which will increase the circulation time of the dye *in vivo* (Xu J. et al., 2022). Due to the ACQ effect, it was screened and found that BSA@IR-817 fluoresced most strongly when the ratio of bovine blood albumin (BSA) to IR-817 was 3:1 and IR-817 concentration was 10 μM, which could be heated up to more than 50°C *in vivo* and *in vitro* when exposed to laser radiation at 808 (1 W/cm^2^ for 5 min), the optimal temperature for thermotherapy, in the subcutaneous construction of a melanoma mouse model, BSA@IR-817 exhibited significant photothermal therapeutic effects under laser irradiation, with a tumor inhibition rate of 99%, and no tumor recurrence was observed even several days after treatment (Wang J. et al., 2023).

### 5.2 Photodynamic therapy

Photodynamic therapy (PDT) is a new non-invasive tumor treatment that uses PSs to transfer energy from light irradiation to oxygen in the tumor, subsequently generating highly cytotoxic ROS that leads to necrosis or apoptosis of the tumor tissue. PSs have been modified into nanomedicines through various materials including peptides and metal ions to prolong blood circulation time and enhance the EPR effect for better tumor accumulation, thereby improving the precision and efficiency of PDT to eradicate tumors (Sun et al., 2017; Adir et al., 2020; Casellas et al., 2022). The nanomedicine, which can be activated and can release PSs on time, can maximize therapeutic efficiency and minimize off-target effects when internalized in targeted cancer cells (Yang et al., 2017). Nonetheless, current photosensitizer nanocarriers still suffer from the disadvantages of complex fabrication, poor dilution stability, low loading capacity, and adverse reactions associated with toxicity, immune and inflammatory responses (Feliu et al., 2016). Therefore, the development of nanomedicines that combine ease of preparation, good biocompatibility, and robust blood circulation and precisely release photosensitizers in cancer cells remains a major challenge.

Due to their excellent photophysical-chemical properties, porphyrins and their derivatives are widely used in PDT and PTT. Porphyrin derivatives for PTT applications generally have large conjugated and aggregated structures and exhibit strong near-infrared absorption and non-radiative excitation efficiency. Enhanced porphyrin delivery at the tumor site is a key issue.

Adenosine triphosphate (ATP) was found as a modifier of porphyrin. Negatively charged ATP can induce the self-assembly of cationic molecules with planar π-systems via electrostatic interactions (Li et al., 2019), so that in aqueous solutions, the co-assembly of cationic porphyrin and ATP can form metal-free helical nanofibers. Porphyrin-ATP nanofibers enhance the delivery of PSs to the tumor site compared to cationic porphyrin alone. Since the concentration of ATP in tumor tissues was much higher than that in healthy tissues, overexpressed extracellular ATP stabilized the porphyrin-ATP NPs in tumor tissues, thereby enhancing the uptake by the cancer cells, and when ATP was biodegraded by an intracellular phosphatase, the enzyme triggered the release of the PSs, which absorb a specific wavelength of the spectrum (with peak values of 405nm or so) and become excited porphyrin. The loss of energy emits red fluorescence, and the transfer of energy to other molecules leads to a phototoxic reaction. Singlet oxygen (^1^O_2_), peroxide, and other free radicals result in lipid membrane oxidization, destroying the membrane structure, impeding transporter function, inhibiting enzyme activity, and causing cellular energy metabolism obstruction, showing tumor treatment effect (Li Z. et al., 2021). N. M. Casellas et al. first generated a novel porphyrin-based monomer, Por (PEG) 4, through hydrophobic forces and π-π interactions between aromatic nuclei, which self-assembled into one-dimensional nanofibers in aqueous medium. When endocytosis, Por (PEG) 4 supramolecular nanofibers selectively accumulate in lysosomes and gradually decompose, thereby releasing and activating porphyrin units, generating ROS and performing fluorescence emission. The results of *in vivo* experiments in animals showed that Por (PEG) 4 exhibited high photocytotoxicity and antitumor efficacy, with IC50 values as low as 13 μM (Casellas et al., 2022).

Apart from efficient and precise delivery to cancer cells, precise guidance of an external light source to irradiate the tumor lesion and determining the time window for treatment are also critical for PDT therapy, and nanotechnology for fluorescence/magnetic resonance (FL/MR) dual-modality imaging-guided PDT is highly desirable in precise and personalized medicine. Metalloporphyrin molecules can be used not only as PS in PDT but also as NIR FL imaging probes, and their phototherapeutic and physicochemical properties can be finely tuned by modulating their central metal species, e.g., Gd3+-porphyrin has the highest proton relaxation efficiency in MR imaging, but poor water solubility and low molecular weights have limited the practical applications and clinical translations of metalloporphyrin. Nanomaterials can deliver metalloporphyrin with significant EPR effects by neglecting their intrinsic solubility. Gd3+-porphyrin-based NPs(Gd-PNPs) was assembled layer-by-layer by Gd-TCPP, PEI and PGA-g-mPEG, and *in vivo* studies demonstrated that Gd-PNPs exhibited excellent biocompatibility, effective tumor accumulation and long residence time at the tumor site as well as excellent FL imaging properties, high longitudinal relaxation (16.157 mM-1 s-1), and good ^1^O_2_ generation properties, thus facilitated FL and T1-MR imaging-guided PDT (Chen et al., 2021).

TME, through the interactions between its complex compositions, has an important impact on tumor development and treatment. M. Y. Zhao et al. developed a TME-responsive, supramolecular NPs (UCNP@MnOx-Hyp) by enabling manganese oxide (MnOx) and the polyhydroxy photosensitizer hypericin (Hyp) to be encapsulated and loaded onto lanthanide-doped upconversion NPs (UCNP). Triggered by GSH within the TME, the Mn^2+^ released and forms Mn^2+^-Hyp, and encapsulates the UCNPs into the crosslinked UCNP@Mn2+-Hyp. The red-shifted uptake rate of the Hyp improves the efficiency of the energy transfer from the UCNPs to the Hyp by a factor of 5.6, and in turn, the cross-linked UCNP exhibited resistance to water quenching, and enhanced luminescence recovery further increased the photodynamic effect, leading to a 2.1-fold increase in therapeutic efficacy at the cellular level and superior therapeutic results *in vivo* (Zhao et al., 2023).

Cancer stem cells (CSCs) are responsible for tumor initiation, progression, metastasis, chemotherapy resistance and relapse (Zhang Z. et al., 2022), research evidence suggests that ROS can control CSC self-renewal and stemness. Compared to chemotherapeutic agents that generate limited endogenous ROS through intrinsic biological pathways, PDT induces a burst of ROS through a photochemical reaction and shows great potential in eliminating CSCs (Kirtonia et al., 2020; Ouyang et al., 2022).

Because unsaturated aliphatic chains can promote the self-assembly of water-insoluble molecules, Q. Wang et al. introduced oleic acid (OA) to couple a hemicyanine scaffold dye (CyOH or SO3-CyOH), which can self-assemble into supramolecular NPs without any exogenous excipient. The supramolecular NP consisting of the CyOH-OA affixation molecular (CyOA NPs) carries positive charges, enhancing cellular uptake and mitochondrial accumulation, whereas supramolecular NPs formed from SO3-CyOH-OA (SO3-CyOA NPs) are negatively charged, and SO3-CyOA NPs are located in lysosomes rather than mitochondria due to their negatively charged surfaces. The correlation coefficient between mitochondria and CyOA was 0.92, which was much larger than the correlation coefficient between lysosomes and CyOA (0.55). CyOA NPs target the mitochondrial complex II succinate dehydrogenase (SDHA) to inhibit oxidative phosphorylation and reverse tumor hypoxia, thereby preserving the endogenous O2 for the subsequent mitochondrial ROS burst, maximizing the killing capacity of cancer cells. Compared to SO3-CyOA NP, which cannot target mitochondria, phototoxicity to breast cancer stem cells (BCSC) was 50.4-fold higher, and in 4T1 and BCSC tumor models, CyOA NPs achieved higher tumor suppression and fewer lung metastatic nodules compared to the clinically used photosensitizer Hiporfin (Wang Q. et al., 2023).

### 5.3 Combination of PTT and PDT

**FIGURE 4:**
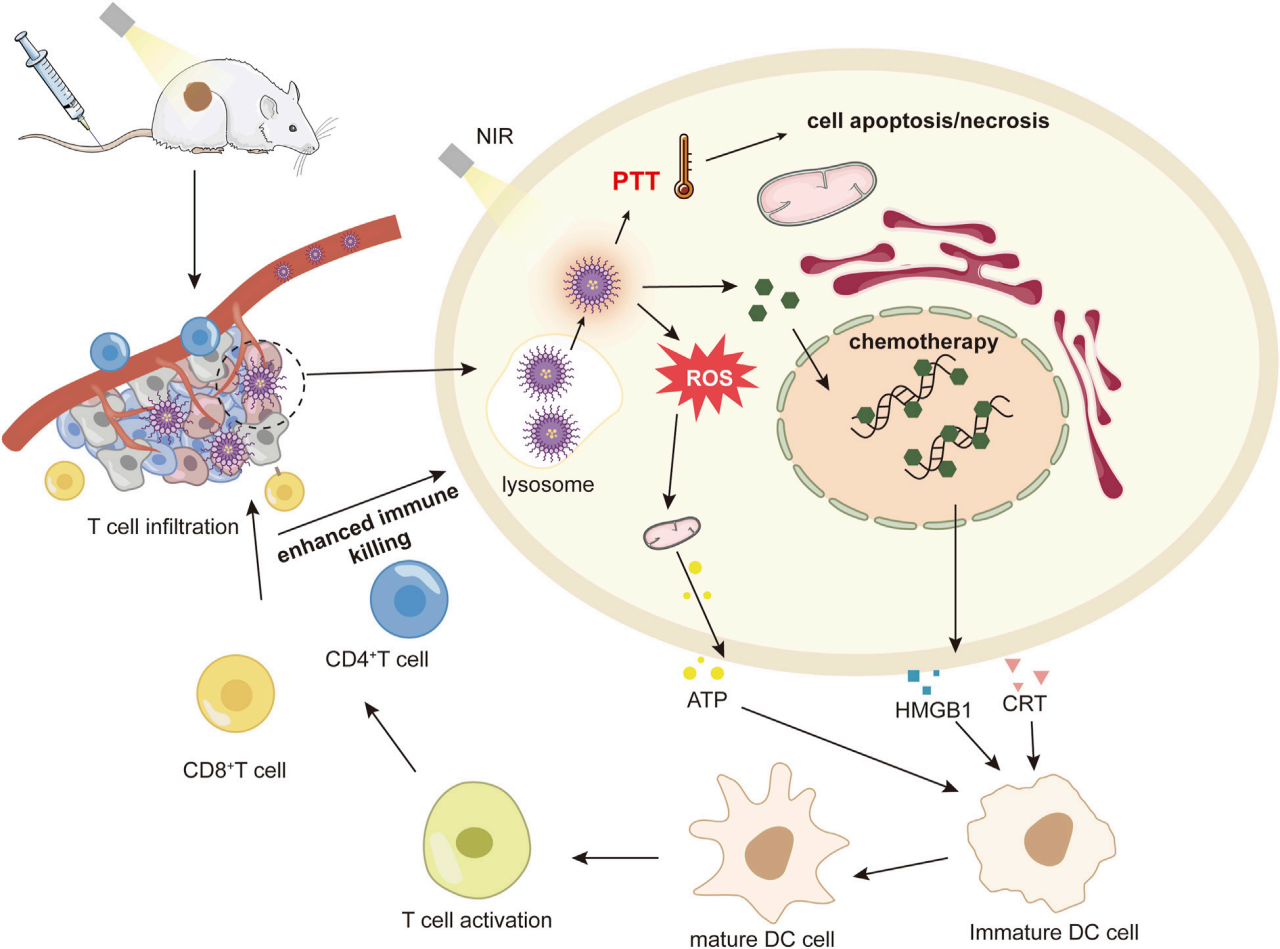
The mechanism of anti-tumor immune response activated by nanomedicine-mediated photochemotherapy.

PDT and PTT are distinct but closely related, with the ROS produced by PDT increasing the capture of laser light by photothermal reagents. The photothermal effect of PTT increases the oxygen supply to the tumor and promotes PDT, especially for some small molecules that have both photothermal and photokinetic properties; synergistic treatment of the two is achieved through a nano-delivery system to create a powerful anti-tumor effect (Figure 4).

Peptides are typically molecularly encoded building blocks capable of subtly manipulating the structure and function of supramolecular materials via the amino acids in the coding sequence. Chang et al. utilized three typical dipeptides, diphenylalanine (FF), dityrosine (YY), and diaspartic acid (DD) to design a series of peptide-PSs couplings, whereas aspartic and tetra-aspartic acids were also included to assess the effect of varying the number of amino acids and investigated their self-assembly and phototherapeutic behavior. The results showed that all the synthesized nanomedicines exhibited high photothermal conversion efficiencies; however, they exhibited different phototherapeutic activities. The PD nanofibers (NFs) exhibited better structural stability than the PDD NFs and PDDDD NFs, and the increase in stability could be attributed to the enhancement of the hydrophobicity effect, while the difference between PYY NFs and PFF NPs can be attributed to enhanced intermolecular hydrogen bonding. The improved structural stability of PD NFs and PYY NFs ensures their selective tumor accumulation, effective intra-cellularization, and high photothermal conversion in the biological environment, leading to tumor ablation without recurrence or detectable side effects. These results suggest that non-covalent interactions can be controlled by encoding amino acid sequences and types to design supramolecular nanomedicines with optimal phototherapeutic activity; however, the specific R and D design requires further investigation (Chang et al., 2022).

Y. Qin et al. successfully constructed discrete Pt6L3 metal clusters (C-DTTP) with high fluorescence efficiency in the NIR-II region by assembling the aggregation-induced emission (AIE)-active four-armed ligand, DTTP, with the 90° Pt receptor, Pt (PEt3) 2 (OTf) 2. C-DTTP exhibits remarkable AIE properties, with emission maxima of up to 1,005 nm. In addition, C-DTTP exhibited enhanced ROS generation and photothermal conversion efficiency compared to the ligand DTTP, which could be attributed to the enhanced inter-systemic crossover and caging structure. The skillful combination of all the desired features in C-DTTP makes it useful in the dual fluorescence/photothermal imaging-guided PDT/PTT synergistic therapy guided by NIR-II with extraordinary performance (Qin et al., 2022).

It is well known that the high intersystem crossover (ISC) efficiency of photosensitizers facilitates the ^1^O_2_ production, and the conventional strategy to promote the ISC process is to enhance the spin-orbit coupling (SOC) of photosensitizers by introducing heavy atoms or special organic parts, however, this approach still has some drawbacks and limitations (Yang et al., 2016). Organometallic Ru- arene complexes offer great advantages owing to their ionic nature and pseudo-octahedral half-sandwich structure (Soliman et al., 2020; Wei et al., 2021; Zhang Z. et al., 2022). However, the inherent drawbacks of low stability and/or poor bioavailability can impair therapeutic efficacy and *in vivo* performance.

Xu et al. reported a NIR-triggered D-A conjugated Ru (II)-arene complex (RuDA) through the coordination bond between a D-A-D chromophore and the Ru(II)-arene moiety, and the resultant complexes can be self-assembled in water via non-covalent interactions into supramolecular vesicles. RuDA47-49 was encapsulated by Pluronic F127 (PEO-PPO-PEO) to enhance tumor accumulation and biocompatibility *in vivo*, forming RuDA NPs. Supramolecular assembly confers intersystemic crossover properties to RuDA, thereby greatly improving ISC efficiency, which is highly beneficial for PDT, and both *in vitro* and *In vivo* experiments showed that RuDA-NPs with good biocompatibility and tumor accumulation exhibited excellent anticancer activity under 808 nm laser irradiation (Xu G. et al., 2022). In addition, H. B. Cheng et al. used boron dipyrromethenes (BODIPYs) dye derivative B4 with long wavelength absorption, high PCE and high photostability, and B4 molecules self-assembled into B4NP (Kubheka et al., 2020; Shi et al., 2020), providing a dual-mode PTT/PDT therapeutic nanoplatform based on single NIR laser excitation with photothermal conversion (η = 6.02%) and ^1^O_2_ generation under 808 nm laser irradiation. By combining B4 with immune checkpoint blockade therapy, this host-object biomarker displacement activation (BDA) strategy may provide opportunities for *in vivo* time-regulated photodynamic and photothermal immunotherapy (Cheng et al., 2022).

## 6 Future prospects

Supramolecular nanomedicine combines the advantages of nanotechnology and supramolecular chemistry, using non-covalent bonding forces to assemble multiple diagnostic and therapeutic molecules, which can not only specifically release drugs in the TME (e.g., weak acidity, specific enzymes, and different redox environments) and external stimuli (e.g., light, ultrasound, and magnetic fields) but can also achieve multiple synergistic therapies, thus providing great possibilities for the precise release of drugs and elimination of drug resistance. Owing to their unique structure, supramolecular drugs have many advantages such as good safety, low toxicity, fewer adverse reactions, high bioavailability, strong drug targeting, low multidrug resistance, good biocompatibility, masking of drug odor, high efficacy, low cost of development, and a high likelihood of success. Many of these drugs are already in clinical use, effectively reducing the side effects of drugs, preventing pain caused by cancer progression and chemotherapy, and providing improved chemotherapeutic methods.

Although supramolecular nanomedicines have made considerable progress in recent years, we are also facing a very tough test and still have a long way to go before clinical translation.

(1) The *in vivo* stability of these supramolecular nanodrugs must be addressed. Non-covalent bonding interaction forces are rich in stimulus responsiveness but also carry the problem of poor stability. Electrostatic interactions are easily disrupted or weakened by metal ions and salts *in vivo*, and charged systems are easily removed by protein adsorption. Hydrophobic interactions also have some interfering factors; for example, the hydrophobic regions of albumin can harbor hydrophobic drugs, which may possess stronger binding capacity than CD/drug complexation. Albumin paclitaxel injection (Abraxane) is a classic example, where paclitaxel can bind well to albumin, improving the water solubility and stability of paclitaxel, and greatly improving the drug’s circulation time (half-life up to 27 h). However, attempts to combine albumin with other drugs have not been ideal, requiring researchers to continue to consider how to solve the problem of intermolecular interactions and stability.
(2) Whether the strategy of designing supramolecular nanodrugs based on the EPR effect is correct is debatable because nanodrugs often have inconsistent efficacy in animal tumor models and human tumors. Tumor EPR effects can lead to increased drug uptake compared to normal tissues, which has been confirmed in both animal tumor models and human tumors. However, nanomedicines may not increase drug accumulation in human tumors compared to free drugs; thus, their clinical efficacy may not be superior to that of free drugs. Moreover, the human EPR is heterogeneous and exists in small molecules, macromolecules, and nanodrugs. Small macromolecules and nanodrugs. Many small-molecule drugs also exhibit an EPR effect in tumors after binding to plasma proteins, leading to no difference in the accumulation of nanodrugs and free drugs in human tumors.
(3) Real functional nanomedicine is yet to be developed. Although there has been much research in basic studies, the field of translation is still relatively traditional liposomes, lipid NPs, nanocrystals, organic and inorganic NPs, polymer micelles, and other applications of these primary levels. Real functional, targeted, and environmentally sensitive nanomedicine has not yet been successfully applied.

In future studies, researchers should combine supramolecular chemistry and polymers to prepare supramolecular polymer nanodrug-carrying systems, fully utilizing the stimulus responsiveness of supramolecules and the stability of polymers. Exploring pharmacokinetics and mechanisms of NPs in the body and optimizing the design strategy are also necessary steps to functional nano drugs in clinical use.
